# Recent Advances in Biodegradable and Biocompatible Synthetic Polymers Used in Skin Wound Healing

**DOI:** 10.3390/ma16155459

**Published:** 2023-08-03

**Authors:** Ruojiao Xu, Yifeng Fang, Zhao Zhang, Yajie Cao, Yujia Yan, Li Gan, Jinbao Xu, Guoying Zhou

**Affiliations:** 1 College of Life Science, Zhejiang Chinese Medical University, Hangzhou 310053, China; 18268922805@163.com (R.X.); fangyifeng2022@163.com (Y.F.); lgkzcyx@163.com (Z.Z.); cyj121123@126.com (Y.C.); yanyujiay@163.com (Y.Y.); hz0113@126.com (L.G.); 2 School of Materials and Energy, Guangdong University of Technology, Guangzhou 510030, China

**Keywords:** synthetic polymer, biodegradable, biocompatible, skin wound healing, fabrication

## Abstract

The treatment of skin wounds caused by trauma and pathophysiological disorders has been a growing healthcare challenge, posing a great economic burden worldwide. The use of appropriate wound dressings can help to facilitate the repair and healing rate of defective skin. Natural polymer biomaterials such as collagen and hyaluronic acid with excellent biocompatibility have been shown to promote wound healing and the restoration of skin. However, the low mechanical properties and fast degradation rate have limited their applications. Skin wound dressings based on biodegradable and biocompatible synthetic polymers can not only overcome the shortcomings of natural polymer biomaterials but also possess favorable properties for applications in the treatment of skin wounds. Herein, we listed several biodegradable and biocompatible synthetic polymers used as wound dressing materials, such as PVA, PCL, PLA, PLGA, PU, and PEO/PEG, focusing on their composition, fabrication techniques, and functions promoting wound healing. Additionally, the future development prospects of synthetic biodegradable polymer-based wound dressings are put forward. Our review aims to provide new insights for the further development of wound dressings using synthetic biodegradable polymers.

## 1. Introduction

Skin is the largest and outermost organ of the body which plays crucial roles in the prevention of microbial invasion, dehydration, and osmoregulation [1]. However, it can be damaged by several factors such as burns, accidents, pathogens, and diseases like diabetes, causing acute or chronic wounds. It is reported that millions of people suffer from acute or chronic wounds every year which imposes a significant burden on public health care [2].

Skin has the ability to heal itself for small-sized wounds. However, the self-repair ability is compromised in the case of large-area skin defects and deep ulcers [3]. In such cases, wound dressings are necessary to cover the wound sides temporarily, avoid secondary injury caused by external stimuli, and trigger the endogenous healing system [4]. Over the past few decades, there have been numerous attempts to develop skin wound dressings [5]. Traditional wound dressings made from cotton or synthetic fibers can help to keep wounds dry but have a limited effect on the acceleration of wound healing. Additionally, dry healing is not conducive to epithelial tissue growth; yet notably, wounds are more prone to mechanical re-injury during dressing changes [6]. Current wound dressings involve various polymer-based systems with multi-functions that have shown promising potential to speed up wound healing [7]. 

Polymers can be divided into natural and synthetic polymers according to their origin. Natural polymeric biomaterials include polypeptides (such as collagen, gelatin, and silk fibroin (SF)), polysaccharides (such as sodium alginate (SA), chitosan (CS), cellulose, and hyaluronic acid (HA) derivatives), and so on [8]. These materials are derived from biological sources which possess excellent biocompatibility and can be degraded by enzymes [9]. Beyond that, their specific molecular structures can assist in wound healing. For example, chitosan contains abundant amino groups which carry positive charges that attract negatively charged red blood cells and proteins, thereby exhibiting a procoagulant effect [10]. Silk fibroin contains a great number of (Gly–Ala–Gly–Ser) amino acid repeat sequences in the crystalline region which may both play a role in biorecognition signaling and promote cell adhesion [11]. Also, HA can bind to the CD44 receptor on keratinocytes and activate a cascade of reactions to promote cell differentiation [12]. Therefore, numerous natural polymer-based biomaterials have been developed as wound dressing materials [13]. However, natural polymers have poor processing properties, low mechanical properties, and fast degradation rates which limit their applications [1,14].

By comparison to natural polymer materials, synthetic polymers possess improved processability and enhanced mechanical properties [15]. Particularly, biodegradable synthetic polymers have emerged as a hot spot for biomedical material development due to their biodegradability, biocompatibility, and functional versatility [16]. Biodegradable synthetic polymers can be degraded through the breaking of molecular chains in vivo; the degradation products can be absorbed or excreted by metabolism without causing secondary damage to human health [17]. Additionally, biodegradable synthetic polymers can be fabricated into different types of materials or products such as gels, nanofibers, and sponges through self-assembly or physicochemical reactions which can mimic the complex structure and function of human skin tissue [18]. Moreover, the structure and properties of the synthetic polymer products can be controlled by varying the process parameters and material composition in order to meet the treatment needs of different types of wounds [15].

Therefore, the aim of this review is to provide an up-to-date overview of applications in the treatment of skin wounds focused on several synthetic biodegradable polymers including polyvinyl alcohol (PVA), polycaprolactone (PCL), polylacti-cacid (PLA), poly(lactic-co-glycolic acid) (PLGA), polyurethane (PU), and polyethylene oxide/polyethylene glycol (PEO/PEG). Furthermore, the future development prospects of skin wound dressings based on synthetic biodegradable polymers are described. 

## 2. Fabrication Techniques Applied in Skin Wound Healing

**Figure 1:**
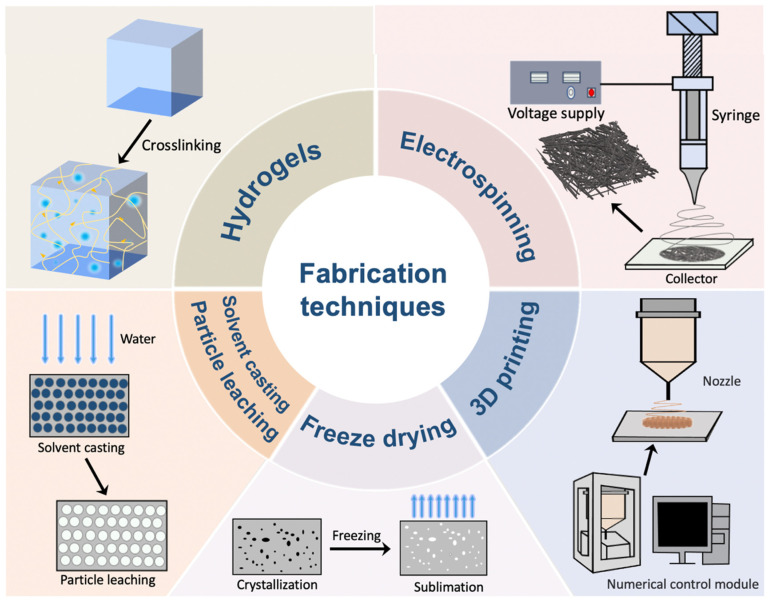
Fabrication techniques for the construction of skin scaffolds.

Depending on the specific applications, different fabrication techniques such as hydrogel formation, electrospinning, solvent casting, freeze-drying, and 3D printing technology have been applied to construct skin scaffolds using various synthetic biodegradable polymers [19,20,21]. The fabrication techniques for the construction of synthetic polymer-based skin scaffolds are summarized in Figure 1.

### 2.1. Hydrogel Formation

Hydrogels are three-dimensional (3D) networks of hydrophilic polymers with tunable physical and chemical properties which can offer a stable and favorable environment to support the wound healing process [22]. Due to the presence of hydrophilic components, when applied on wounds hydrogels can hold large amounts of water, maintaining the moisture environment at the wound site but also absorbing the wound exudates and preventing infection [23]. 

According to the molding method, hydrogel fabrication techniques can be classified into the template method (using a template to control the shape of the hydrogel) [24], 3D printing (stacking hydrogel materials layer by layer to form complicated 3D structures by manipulating the position of the printing nozzles) [25], solvent casting (pouring a hydrogel precursor solution into a flat container to form hydrogels by evaporation of the solvent) [26], and hydrogel self-assembly (formation of hydrogel by controlling intermolecular interactions and assembly behavior) [27]. Additionally, the mechanical and degradable properties of the hydrogels can be precisely regulated by varying the preparation conditions such as the degree of crosslinking and molecular weight of the polymers to meet the needs for the treatment of different skin defects [28]. Furthermore, hydrogels can be used as drug carriers and endow sustained release of the loaded drugs which can improve the retention time and thus exert a better treatment outcome for skin healing [29]. Therefore, hydrogels have become considerable candidates for medical wound dressing in recent years [30].

Although hydrogels have shown great potential as wound dressing materials, they still have limitations. To develop hydrogels that more intelligently meet the needs of skin wounds, novel hydrogel systems with the properties like self-healing, high tissue adhesion, stimulus-response, conductivity, and wound monitoring features are being developed [31,32].

### 2.2. Electrospinning

Electrospinning is a technique based on electrical charges to form polymer microjets in a high-voltage electric field followed by rapid solidification into continuous nanofiber structures in the air [33]. These nanofibers have similar microstructures like the extracellular matrix which can mimic the structure and function of skin. Compared with traditional dressings, electrospun nanofibers have larger specific surface areas, ensuring the air exchange with the outside world while preventing the invasion of bacteria [34]. In addition, it is feasible to adjust parameters such as polymer composition, solution concentration, applied voltage, distance, etc., to fabricate fibers conducive to cell adhesion and proliferation [35]. Furthermore, due to the biocompatibility and degradability of the polymers, fabricated nanofibers can be absorbed by human tissues without causing infection which is beneficial for skin wound healing [36].

With the development of the electrospinning technique, devices such as coaxial nozzles, parallel nozzles, and multi-nozzles have emerged to meet the needs for a diverse range of structural nanofibers, further optimizing its applications in skin injury treatment [37,38,39].

### 2.3. Freeze Drying

Freeze-drying is a low-temperature drying process in which the solution is converted to a solid state under low temperatures and low pressure, followed by the removal of the frozen solvent (usually water) through a process of sublimation [40]. In such a way, freeze-drying can avoid the denaturation of substances (like enzymes and proteins) which might be caused by the heating procedures in conventional drying methods and thus can maintain the original texture as much as possible [41]. The freeze-drying technique can customize the pore structures of the scaffolds by adjusting the parameters of the starting polymer solution including the concentration and viscosity as well as the processing parameters like the freezing rate, drying conditions, and refrigerant [42]. Drugs can be loaded into the pores of the scaffold and the release rate can be regulated by the pore structures to achieve continuous and stable drug release [43]. Furthermore, the porous structure enables efficient oxygen and nutrient transport, maintaining a suitable microenvironment to promote wound healing [44]. However, the long processing time and uneven heat transfer during the freeze drying process might lead to heterogeneity of the fabricated scaffolds which is one of the drawbacks needed to be overcome for this technique [45].

### 2.4. Solvent Casting/Particle Leaching

Solvent casting/particle leaching is a technique for preparing porous scaffolds by dissolving different particles such as sodium sugar particles [46], chloride particles [47], ice particles [48], and so on. The pore size and porosity of the scaffolds can be accurately controlled by dissolving different sizes and numbers of particles [49]. It has been confirmed that the pore size of scaffolds can affect cellular behaviors like cell adhesion and proliferation and thus affect the wound-healing process [50]. Compared to other methods, solvent casting/particle leaching does not require specialized equipment, which, coupled with the porous structure it produces, makes it a suitable technique for skin tissue engineering applications [51]. However, the porous structures of the scaffolds can reduce the mechanical properties. This can be strengthened by using different solvent systems or by adding different plasticizers including glycerin, polyethylene glycol, and sorbitol [52]. In addition, new strategies to improve the pore uniformity and pore interconnection of the scaffolds should be developed for targeting to attain an improved efficiency for promoting wound healing using the solvent casting/particle leaching technique.

### 2.5. Three-Dimensional (3D) Printing

Three-dimensional (3D) printing is a technique that fabricates three-dimensional objects in a layer-by-layer manner using digital models, which has also been proved as a feasible technique for skin injury treatment [53]. The implementation of this technology initially involves scanning or measuring the skin area followed by designing a 3D model based on the resulting data [54]. Next, bioink is deposited to build sequential 3D layers resembling human skin [54]. It is worth noting that biocompatible and degradable polymers are commonly used as scaffold materials in 3D bioprinting [55]. Thanks to the precise control of the morphology and structure of the material by 3D printing, skin scaffolds can be customized based on individual characteristics [56]. Nevertheless, they currently cannot achieve both high cell density and detailed resolution; further development of the technique to overcome these drawbacks is necessary [57,58].

It is worth noting that choosing between different fabrication techniques should be determined by the different needs of the types of skin wounds. Therefore, the combination of multiple fabricating techniques can take advantage of different techniques to obtain a superior wound dressing material. For example, a nano-fiber mesh grid made by electrospinning can be used as the top layer of the scaffold while hydrogels can be embedded to mimic the dermis. This combination of techniques enables the scaffold to better mimic the multilayered structure of skin which can overcome the limitations of a single fabricate technique [57].

## 3. Synthetic Polymer-Based Skin Scaffolds 

By contrast to natural polymers, degradable synthetic polymers can be designed and synthesized to achieve more precise control of the physical and chemical properties, finding various applications in wound dressings, drug delivery materials, tissue engineering scaffolds, and so on [59]. When applied to skin wounds, these polymer-based wound dressings can not only provide support and protection to the wound sites but also serve as carriers for cells and growth factors, promoting cell proliferation and skin tissue repair [60]. Next, we will introduce the applications of the degradable synthetic polymers applied in skin wound healing, including PVA, PCL, PLA, PLGA, PU, and PEO/PEG which are all approved by the US Food and Drug Administration (FDA) to be used in biomedical applications [61,62,63,64]. Examples of these scaffolds with different compositions, forms of scaffold, degradation times, and main effects related to diabetic wound healing are presented in Table 1.

### 3.1. PVA

**Figure 2:**
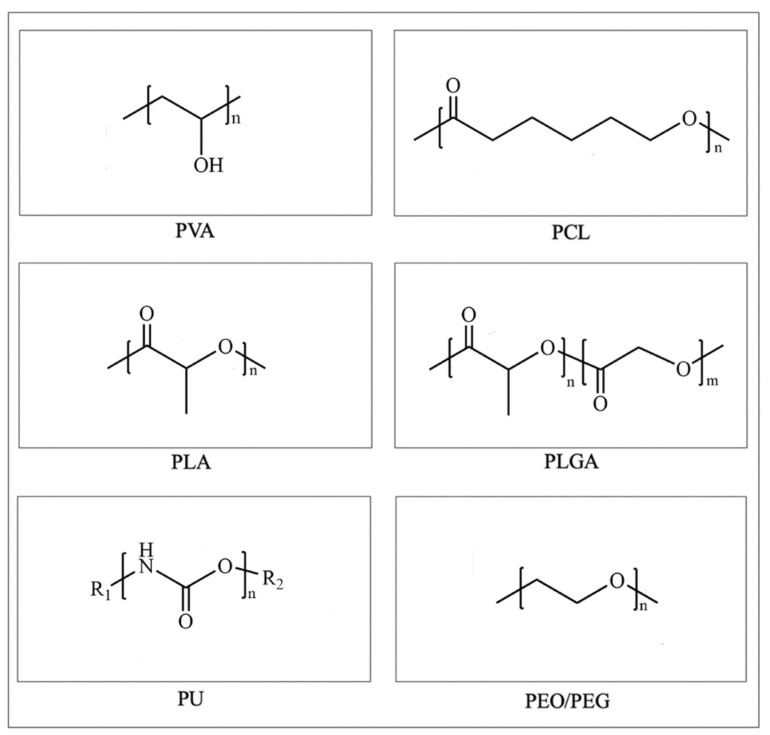
The chemical structures of PVA, PCL, PLA, PLGA, PU, and PEO/PEG.

Polyvinyl alcohol (PVA) is a non-toxic and water-soluble polymer made from the polymerization and alcoholysis of vinyl acetate [65]. It possesses a represented chemical formula of (C_2_H_4_O)n, as shown in Figure 2. The large amount of hydrophilic groups in PVA endow it with excellent hygroscopicity and allow for its biodegradation into water and carbon dioxide [66]. The safety of PVA implants in medical applications was demonstrated by replacing diseased spinal cores in a primate baboon model [67]. Recently, a breakthrough study published in Nature found that PVA can replace human serum albumin (HAS) to support hematopoietic stem cell (HSC) growth and maintain the stemness, for the first time achieving a thousand-fold expansion of HSCs in vitro [68]. This reveals the enormous potential of synthetic polymers like PVA in biomedical applications. 

PVA has also been developed as a biomedical material for skin wound healing owing to its excellent biodegradability, biocompatibility, and non-toxicity. For example, PVA sponges serve as clinical hemostats for diverse operations to reduce the risk of postoperative bleeding [69]. In addition, PVA can be crosslinked by various physical, chemical, or radiation methods to form PVA-based hydrogel dressings [70]. Nonetheless, a mass of hydrophilic hydroxyl groups in the molecular chain of PVA results in a thicker water layer at the exudate site which may lead to bacterial infection [70,71]. This weakens its curative effect in skin lesions and the modification of PVA with natural polymers or bioactive compounds is promising to compensate for these limitations.

**Figure 3:**
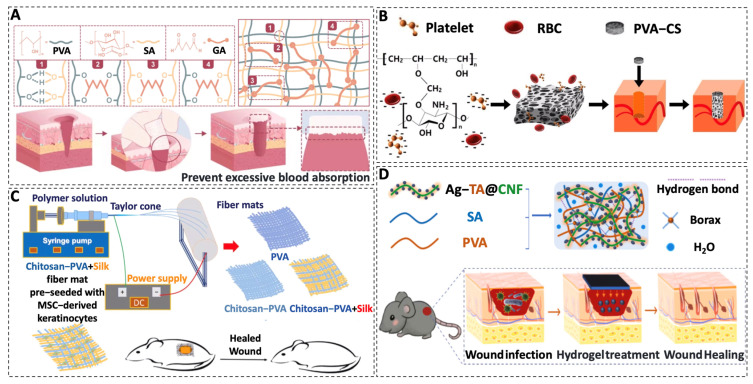
Combination of PVA and natural polymers as well as other add-ons for the construction of skin tissue engineering scaffolds. (**A**) PVA and SA were crosslinked by GA to establish a sponge that swells at the wound in response to hemostasis and prevents excessive blood absorption after thrombosis. Reprinted with permission from Ref. [73]. 2022, Elsevier. (**B**) PVA–CS sponge acts to promote hemostasis by attracting negatively charged blood cells and exerting pressure on bleeding vessels. Reprinted with permission from Ref. [74]. 2019, Royal society of chemistry. (**C**) Nanofiber mats containing PVA, CS, and SF were fabricated by electrospinning and applied to a rat full thickness wound model after inoculation of bone marrow MSCs which significantly promoted wound healing [75]. (**D**) Hybrid hydrogels composed of Ag-loaded nanoparticles and TA-modified CNF with PVA and SA were used to treat wounds in rat wound models by anti-infection. Reprinted with permission from Ref. [77]. 2021, Elsevier. Reproduced with permission [73,74,75,77]. Abbreviations: PVA—polyvinyl alcohol; SA—sodium alginate; GA—glutaraldehyde; RBC—red blood cell; CS—chitosan; SF—silk fiber; MSCs—mesenchymal stem cells; TA—tannic acid; CNF—cellulose nanofibrils.

The combination of PVA with natural polymers such as sodium alginate (SA), chitosan (CS), gelatin, and fibrin can endow PVA with improved water absorption capacity and enhanced cell adhesion/proliferation as well as improved wound healing effects [72]. For example, Li et al. prepared a composite sponge of PVA and sodium alginate with ordered microchannels and disordered porous structure by the 3D printing sacrificial template and freeze-drying method [73]. Compared to the traditional PVA hemostatic sponge, the PVA/SA sponge has a stronger water absorption capacity and improved mechanical property which can accelerate blood convergence and promote coagulation, revealing a new avenue for the development of hemostatic sponges [73] (Figure 3A). In another study, CS was modified with PVA to produce a PVA/CS sponge through foaming and cross-linking reactions. The PVA/CS sponges showed high biocompatibility, excellent hemostatic performance, and enhanced wound healing effects [74] (Figure 3B). Furthermore, the incorporation of CS and silk fiber (SF) into the PVA electrospun nanofibers resulted in improved mechanical properties, enhanced cellular attachment and proliferation, and accelerated skin regeneration, showing great potential as a skin substitute for the repair of injured skin [75] (Figure 3C). In our work, a novel PVA–fibrin composite scaffold is fabricated by an emulsion templating method, showing facilitated angiogenesis, am accelerated wound healing rate, and high potential as a skin substitute [76].

Currently, numerous studies have shown that the incorporation of bioactive compounds such as antibacterial agents, growth factors, and herbal extracts can enable scaffolds with improved effects in supporting wound healing [78,79]. For example, the antibacterial silver nanoparticles were loaded to a lignocellulose, sodium alginate, and PVA-based nanocomposite hydrogel (ATC/SA/PVA) [77]. The composite hydrogel exhibited a broad-spectrum antibacterial effect and promising wound healing results [77] (Figure 3D). In another study, it was revealed that the self-degrading traits of SA and lignin could accelerate the degradation of PVA films [80]. It is known that growth factors induce a range of intracellular biochemical activities and hold a key position in regulating the wound healing process [81]. Some growth factors such as platelet-derived growth factor (PDGF), fibroblast growth factor (FGF), and transforming growth factor (TGF) have already been used in clinical trials. However, when applied individually, the growth factors showed a short half-life, spread rapidly over the wound, and were degraded by active proteases [82]. Therefore, researchers loaded various growth factors onto polymer scaffolds in order to prolong the lifespan of the growth factor but also improve the function of the scaffolds. For example, Bahadoran et al. prepared a skin scaffold of PVA/SA hydrogel combined with microspheres encapsulated with basic fibroblast growth factor (bFBG) by the freeze-thawing method and the fabricated scaffold effectively promoted skin tissue regeneration [83]. 

### 3.2. PCL

Polycaprolactone (PCL) is an organic polymer obtained from the ring-opening polymerization of ε-caprolactone monomers [84]. As shown in Figure 2, the presence of ester groups endows PCL with degradable properties. PCL can be hydrolytically cleaved and converted into oligomeric fragments which are subsequently phagocytosed and digested by macrophages [85]. The efficiency of PCL degradation can be modulated by the adjustment of the molecular weight, shape, autocatalysis, and so on [86]. However, the degradation rate of PCL is much slower compared to other biodegradable synthetic polymers which makes PCL an ideal candidate for long-term drug delivery systems such as the contraceptive implant [87]. For example, the PCL-based subdermal contraceptive implant Capronor^®^ demonstrated long-term security in the drug release system in earlier trials in the 1980s [88]. Nowadays, the remarkable properties of PCL, such as its ease of processing, good balance between mechanical properties, biodegradability, and biocompatibility make it suitable for various tissue engineering applications such as bone, cartilage, nerve, cardiovascular tissue, skin, etc. [89,90,91,92].

PCL has shown immense potential in the fabrication of scaffolds for the acceleration of wound healing. Particularly, the slower degradation rate allows PCL good candidate for the delivery of growth factors, antibacterial reagents, and other bioactive reagents in order to enhance the biocompatibility, antibacterial property, and wound healing-promoting effects. For example, epidermal growth factor (EGF) was covalently immobilized on PCL and collagen nanofibers which not only improved the absorbency and biodegradability of PCL nanofibers but also markedly upregulated the expression of the skin-related gene loricrin [93]. An experiment incorporated curcumin into an electrospun nanofiber scaffold of PCL/SF and PVA/SF to achieve targeted transport and biphasic release of curcumin to promote wound healing through a variety of cellular pathways and molecular targets [94,95]. It was also revealed that PCL/SF/curcumin scaffolds require a longer degradation time than PVA/SF/curcumin scaffolds, thus facilitating long-term targeted drug delivery to the wound [94]. This can be attributed to the fact that the molecular structure of PCL contains a large number of nonpolar groups such as methylene and methyl groups which do not interact strongly with hydrogen bonds and exhibit strong hydrophobicity [96]. The amoxicillin-loaded PCL and gelatin bilayer nanofiber scaffold demonstrates resistance to bacterial growth in disk diffusion assays and preclinical evaluation reveals its potential for healing wounds [97].

However, highly hydrophobic PCL has fewer binding sites for cell adhesion, migration, proliferation, and differentiation during degradation [98]. To alleviate this defect, the researchers added gum tragacanth (GT) for modification. The MTT and SEM results showed that the scaffold significantly improved the adhesion and value-added properties of NIH3T3 fibroblasts [99]. However, the high viscosity of the GT solution and the mutual repulsion of the anions on the GT molecular chain pose a challenge for electrostatic spinning [100]. Zahra et al. further optimized this material and prepared PVA/PCL/GT hybrid nanofibers by the dual nozzle electrospinning process [101]. The presence of PVA and GT accelerated the degradation of PCL and the swelling capacity they provided kept the wound environment moist, thus accelerating wound healing [101]. 

**Figure 4:**
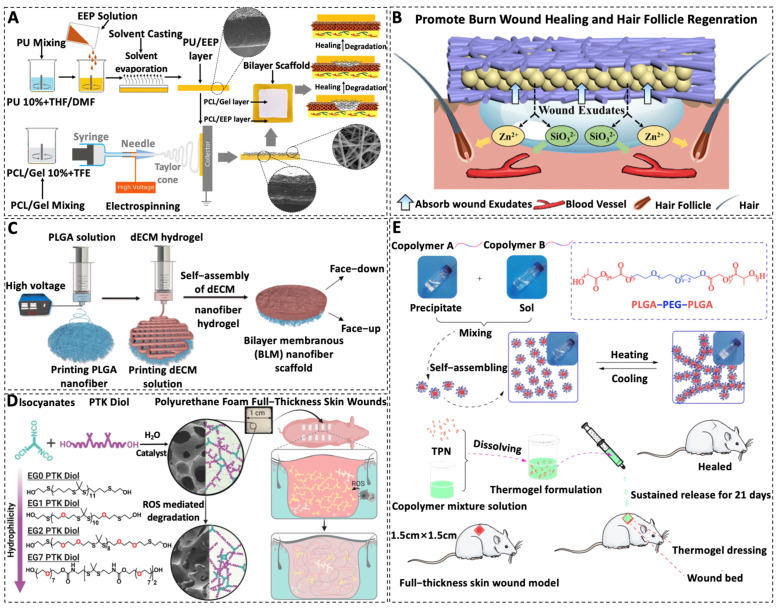
Examples of polymeric constructs for skin tissue engineering scaffolds. (**A**) Double-layer scaffold for wound healing by solvent casting of PU and EEP as an outer dense membrane while electrospun PCL and gelatin nanofibres served as an inner membrane [102]. (**B**) A sandwich-structured wound dressing with Janus membrane property was fabricated by using hydrophilic zinc silicate bioceramics and hydrophobic PLA which showed exudate absorption property and promoted hair follicle regeneration and wound healing through the release of Zn^2+^ and SiO3^2−^ ions [103]. (**C**) The combination of a 3D printed outer membrane made of PLGA nanofibers and an inner membrane composed of dECM nanofibers was employed to inhibit the formation of scar tissues. Reprinted with permission from Ref. [104]. 2022, copyright owner’s name. (**D**) Reactive liquid molding of isocyanates and PTK diols produces PTK-UR foams with tunable properties and interconnected pores suitable for cell infiltration and the repair of skin wounds. Reprinted with permission from Ref. [105]. 2022, American association for the advancement of science. (**E**) Insoluble copolymer A mixed with water-soluble copolymer B self-assembles into micelles, forming an in situ thermogel with a percolated micelle network upon heating; the TPN-loaded polymer solution forms an in situ hydrogel for wound treatment. Reprinted with permission from Ref. [106]. 2019, Springer. Reproduced with permission [102,103,104,105,106]. Abbreviations: PU—polyurethanes; EEP—ethanolic extract of propolis; PCL—polycaprolactone; THF—tetrahydrofuran; DMF—dimethylformamide; Gel—gelatin; TFE—2,2,2-trifluoroethanol; dECM—decellularized extracellular matrix; BLM—bilayer membranous; PTK—polythioketal; UR—urethane; ROS—reactive oxygen species; EG—ethylene glycol; TPN—teicoplanin; PLGA—Poly(lactic-co-glycolic acid); PEG—polyethylene glycol.

In order to overcome the shortcomings of one-layer wound dressings with individual characteristics, bilayer wound dressings composed of two layers with different properties have gained much attention. In the study by Eskandarinia et al., PCL and gelatin nanofibrous scaffolds served as the inner layer to emulate the structure of ECM while a dense membrane formed by polyurethane (PU) and ethanolic extract of propolis (EEP) was applied as an outer layer to protect the wound from mechanical stress and bacterial infection, demonstrating a high potential candidate for biomedical applications [102] (Figure 4A). 

### 3.3. PLA

Polylactic acid (PLA) was originally synthesized in 1932 by Wallace Carothers through condensation polymerization of lactic acid under vacuum [107]. Made from bio-based renewable materials, PLA is biodegradable and recognized as an eco-friendly material [108]. The degradation of PLA in vivo starts with the breakage of ester bonds to lactic acid followed by the oxidation of lactic acid to pyruvate via lactate dehydrogenase which enters the tricarboxylic acid cycle and releases energy [109,110]. Owing to the rapid prototyping, good biodegradability, suitable physicochemical properties, and biocompatibility, PLA becomes a leading material used in various medical applications such as rapid manufacturing of medical equipment, tissue engineering, and drug delivery systems [111]. 

Despite so many advantages, PLA-based artificial skins have drawbacks of brittleness, limited rheological properties, and viscoelasticity when compared to natural skin tissue [112]. It is known that PCL is a ductile semi-crystalline polymer which has been proven to effectively improve the rheological properties of PLA and enhance the ductile deformation of PLA fibers [113]. Based on this, Kobsa et al. designed a PLA/ PCL hybrid electrospun scaffold which was found to possess improved structural morphology as well as enhanced cell attachment and proliferation compared to the PLA scaffold [114]. Furthermore, the PLA/ PCL hybrid scaffold was functionalized and used for the delivery of a plasmid encoding keratinocyte growth factor (KGF) which was found to aid the highly efficient control of KGF delivery and significantly accelerated wound healing in a murine full-thickness defect model [114]. 

In addition, the hydrophobic nature of PLA is not conducive to cell adhesion and thus methods for the hydrophilic modification of PLA scaffolds are needed [115]. Modification of the synthetic polymer-based scaffolds with natural products is one of the most effective methods [116]. For example, PLA scaffolds optimized with gelatine showed improved hydrophilicity and cell compatibility [115]. Yin et al. fabricated electrospun PLA/gelatin/SF composite nanofibers which were identified to have increased spinning capacity, enhanced cell growth, and improved potential for use as tissue engineering scaffolds after the incorporation of gelatin and SF [117]. In another study, hydrophilic zinc silicate bioceramics (Hardystonite, ZnCS) were combined with PLA to constitute a sandwich-structured wound dressing [103]. The Janus membrane exhibited excellent exudate absorption properties and improved hair follicle regeneration and wound healing abilities due to the synergistic effects of the released Zn^2+^ and SiO3^2−^ (ZnCS) [103] (Figure 4B). 

Furthermore, synthetic polymers do not have inherent anti-inflammatory properties but can serve as drug delivery systems to release anti-inflammatory drugs to improve the performance of wound healing [118]. In one study, non-steroidal anti-inflammatory drug ibuprofen was loaded in PLA nanofibers to create scaffolds for the treatment of acute and chronic wounds [62]. The scaffold was found to promote human skin cell viability and proliferation and to reduce wound contraction in vivo. Thereafter, the scaffold was inoculated with human epidermal keratin-forming (HEK) cells in the top layer and human dermal fibroblasts (HDF) in the bottom layer to mimic natural skin which was found to induce vascular growth, showing promising applications in wound dressing materials [62].

### 3.4. PLGA

Poly(lactic-co-glycolic acid) (PLGA) is a polymer made from the irregular polymerization of lactic acid (LA) and glycolic acid (GA). It has the advantages of both PLA and PGA and is also a biodegradable synthetic polymer [119]. In the moist environment of wounds, the ester linkage of PLGA can be hydrolyzed into small fragments which can be excreted by human metabolism [120]. The degradation rate of PLGA can be altered by adjusting the ratio of LA to GA. Generally, the degradation rate increases as the GA content increases due to the more hydrophilic nature of GA than LA [61]. The excellent controllable degradability and tunable physicochemical properties make PLGA an ideal candidate for drug delivery systems [121,122,123] as well as scaffold matrices for various tissue engineering applications [61,119,120].

Despite the superior advantages concerning PLGA-based scaffolds, they are hydrophobic and semi-permeable which means they do not have the capacity to absorb exudates or provide a moist microenvironment when used as wound dressing materials [124]. Rather, they can act as an outer layer of the dressing to protect against bacterial invasion while other scaffold matrices like hydrogels can act as the beneath layer which can promote cell proliferation and wound healing efficiency. By using this strategy, a bilayer membrane (BLM) scaffold consisting of an outer PLGA membrane layer and a lower alginate hydrogel layer was fabricated using 3D printing technology to mimic the epidermis and dermis of skin [125]. It was found that the BLM scaffold resulted in the highest levels of skin regeneration by increasing neovascularization and collagen I/III deposition. In another study by the same group, a decellular dermis matrix (dECM) nanofiber scaffold was used as the lower layer while the PLGA layer was still used as the outer layer [104] (Figure 4C). The results showed that the PLGA-dECM BLM can inhibit the proliferation of hyperplastic scars by the inhibition of collagen fiber deposition and angiogenesis.

On the other hand, PLGA can be combined with other bioactive components such as natural polymers, growth factor inhibitors, and antibacterial species to acquire high-performance PLA composites [126,127,128]. Nanofibrous films based on PLGA and natural polymers normally offer the benefits of both and CS/PLGA scaffolds have shown appealing applications in skin tissue engineering [129,130]. Compared to pure PLGA, the CS/PLGA copolymer provided more hydrophilic properties and the nanofibers manufactured revealed higher cell viability and proliferation results [131]. The scarless treatment eliminates the burden on patients in comparison to suppressing the growth of scarring. Zhang et al. constructed hydrogel capsules with the timely pulsed release of TGFβ inhibitors which resulted in scarless healing in an animal model [132]. Earlier research exploited the disruptive role of silver ions on microorganisms and integrated PLGA and silver nanoparticles to make antimicrobial films based on a solvent casting method [133]. Besides silver ions, copper ions were also found to stabilize hypoxia-inducing factors (HIF-1α), imitate hypoxia, and promote angiogenesis [134]. Based on this, a PLGA bioactive glass dressing was loaded with copper ions and found efficient management of full-thickness skin defects [134].

### 3.5. PU

Polyurethane (PU) contains repeating structural units of the carbamate group (-NH-COO-) in the molecular chain (as shown in Figure 2) which is synthesized from isocyanates and hydroxyl groups of polyols in a stepwise polymerization reaction [135]. PU serves a diverse medical application owing to its excellent biocompatibility and malleability [136]. In 1960, Braunwald first implanted a PU heart valve into the human body [137]. Since the late 1990s, PU has been used as a biomaterial for bone and cartilage repair [138].

In recent years, PU-based wound dressings have shown great potential due to their easy access to the required biological, mechanical, and physico-chemical characteristics [139]. One of the polyurethane foams, NovoSorb^®^, has been commercialized to support wound healing during surgery [140]. However, polyurethane degrades slowly in the body and lacks a specific degradation pathway [141]. In this regard, it can be modified by the introduction of functional groups, nature, other synthetic copolymers, etc., to meet the requirements for skin tissue engineering scaffolds [142,143].

Compared to the individual PU materials, the combination of natural polymers, such as lignin, endows them with improved mechanics and biodegradability [144,145]. Lignin is rich in hydroxyl groups that can react with isocyanate groups to form carbamate bonds [146]. In another study, Li et al. exploited the phenolic hydroxyl groups in lignin as a reducing and capping agent of silver ions to improve the antibacterial properties of the scaffold [147]. Compared to the commercial PU dressing Tegaderm^TM^, the lignin polyurethane/Ag composite foam displayed markedly accelerated wound healing efficiency [147].

The breakage of chemical bonds during PU degradation releases negatively charged molecules such as carboxyl, phenol, and aldehyde groups which in turn form negatively charged ions or entities that cause acidification of the surroundings [148,149]. Combining PU with natural polymers can alleviate the side effects of acid. Hyaluronic acid has been reported to alleviate inflammation by regulating environmental balance in the body and inhibiting the production of free radicals [150]. Wang et al. prepared a hyaluronic acid-waterborne polyurethane (HA-PU) gel which showed good self-healing properties, reduced immune inflammation, and enhanced angiogenesis and hair follicle regeneration [151]. In the study by Patil et al., polythioketal (PTK) was applied to achieve reactive oxygen species (ROS)-dependent degradation of the PU foam dressings without producing acidic by-products [105] (Figure 4B). Compared to NovoSorb, wounds managed by this scaffold resulted in higher wound healing scores characterized by lower inflammation and higher reepithelialization [105].

However, most of the polyols and isocyanates used for the polymerization of PU originate mainly from petroleum which can speed up the depletion of petrol [142]. Therefore, one of the important directions is focused on the introduction of isocyanate-free polyurethanes, biomass chain expanders, and bio-based polyols to achieve the sustainable development of PU [152].

### 3.6. PEO/PEG

Polyethylene oxide (PEO) and polyethylene glycol (PEG) share the same carbon chain backbone. The difference lies in their synthesis method and the molecular weight. The promising biocompatibility, non-immunogenic, and protein resistance ability of PEG make it suitable for applications in drug delivery, cell culture, and tissue repair [153]. 

PEG-based wound dressings have been proven to hold promising potential for the acceleration of wound healing. Cu^2+^/CS/PEG films have shown enhanced mechanical properties and better pro-healing performance compared with individual CS [154]. This can be attributed to the cross-linking ability of PEG with CS as well as the antimicrobial effect of Cu ions. Similarly, Asghari et al. exploited the excellent electrospinnability of PEO to address the difficulty in processing of CS [155]. The synthesized nanofibers could mimic the ECM structure which is essential for the growth of distinct cells in trauma [155]. PEG is one of the most used hydrophilic blocks to construct block copolymers. In a study by Xu et al., PLGA–PEG–PLGA triblock copolymers with varying ratios of PEG/PLGA were synthesized and used to construct blend thermogels with adjustable gelation performance, showing promising effects in the acceleration of wound healing in rats with a fully incised layer [106] (Figure 4E).

Notably, PEG lacks degradable functional groups and is not degradable under normal physiological conditions; excessive use of PEG can lead to the development of anti-PEG antibodies in humans [156]. In this regard, biodegradable PEG polymers can be synthesized by introducing functional groups into the PEG skeleton or by using functional epoxides [157]. Hu et al. synthesized degradable PEG-like polymers by controlled ring-opening polymerization of macrocyclic crown ether lactones [158]. Golba et al. used 1,2,4-triazoline-3,5-diones (TADs) to introduce functional groups along the PEG backbone, resulting in ethers containing hemiamine ether-type linkages which can be hydrolyzed in an aqueous environment [159]. These functionalized PEGs with biodegradability are expected to extend their applications in biomedical fields.

**Table 1:** Summary of various biodegradable and biocompatible synthetic polymer-based scaffolds used in the treatment of wounds.

Polymers	Form of Scaffold	Composition	Main Effects	Degradation Time	References
PVA	Sponge	PVA–SA	Improve hemostatic efficiency	-	[73]
	Sponge	PVA–CS	Procoagulant, high biocompatibility, increased reepithelialization, and reduced granulation tissue	-	[74]
	Electrospun fibers	PVA–CS–SF	Beneficial to cell adhesion and proliferation and stimulate wound healing and skin tissue regeneration	At 16 days, the weight loss rate was nearly 70 percent	[75]
	Hydrogels	PVA–SA–ATC	Antibacterial to prevent wound infection, excellent biocompatibility, and absorb blood and tissue exudate	-	[77]
	Hydrogels	PVA–SA–Microspheres	Antibacterial and promote cell proliferation, epithelialisation, and collagen deposition	Degradation at the wound site occurred within 3 to 4 weeks	[83]
	Electrospun fibers	PCL–Collagen–EGF	Promote cell proliferation and differentiation and significantly up-regulate the expression of skin-related genes loricrin	The wound was degraded by 30% in 7 days	[93]
	Electrospun fibers	PCL–SF–CU	Strong mechanical strength accelerates wound healing	Close to complete degradation within 14 days	[94]
PCL	Electrospun fibers	PCL–GT	Antibacterial and enhance fibroblast adhesion and proliferation	The nanofiber scaffolds retained their morphology after 30 days	[99]
	Electrospun fibers	PCL–PVA–GT	Antibacterial and enhance cell proliferation	After 15 days, the hybrid nanofibers were slightly degraded and after 30 days the nanofibers were more expansive	[101]
	Electrospun fibers	PCL/Gel–PU/EEP	The outer layer is antibacterial and protects the wound while the inner layer promotes cell adhesion and proliferation	After 28 days, only 1.9% weight loss was observed on the PU/EEP membrane and 76% reduction in the PCL/gel scaffold	[102]
PLA	Electrospun fibers	PLA–PCL–KGF	The rate of peripheral epithelial reformation, keratinocyte proliferation, and granulation reaction were improved	Degradation was observed between day 14 and 28	[114]
	Electrospun fibers	PLA–Gelatin	Enhance cell adhesion and proliferation	After ten weeks, there was significant weight damage	[115]
	Electrospun fibers	PLA–SF–Gelatin	Promote cell proliferation	Significant degradation occurred one month after subcutaneous implantation	[117]
	Electrospun fibers	PLA–Ibuprofen	Promote the growth and reproduction of human epidermal keratinocytes and human dermal fibroblasts and support angiogenesis	The scaffolds were slightly degraded after 14 days on the wound	[62]
PLGA	3D printed bilayer membrane	PLGA–Alginate	Increase neovascularization and promote collagen deposition	After four weeks, less than 60 percent of its weight remained	[125]
	3D printed bilayer membrane	PLGA–dECM	Inhibit collagen fiber deposition and angiogenesis and inhibit hypertrophic scar formation	After four weeks, the degradation rate was close to 70%	[104]
	Solvent cast nanocomposite films	PLGA–Ag	Antibacterial	The quality remained unchanged for 25 days and decreased significantly after 1 month	[133]
	Electrospun fibers	PLGA–CS	Promote fibroblast attachment and proliferation	At 56 days, mass loss is greater than 20%	[131]
PU	Foams	PU–Lignin–Ag	Antibacterial and absorb wound exudate	In the alkali-methanol solution system, it was completely degraded from the original solid to the clarified solution at 60 °C for 5 h	[147]
	Foams	PU–PTK	Promote extracellular matrix production and re-epithelialization and relieve inflammation	It was incubated in 20% H_2_O_2_/0.1M CoCl_2_ solution and completely degraded within 20 days	[105]
PEO	Electrospun fibers	PEO–PCL/CS	Antibacterial and cytocompatible	The scaffolds were significantly degraded within 28 days and the earliest was 7 days	[155]
	Hydrogels	PEO–CS–Cu	Antibacterial and promote cell adhesion	The membrane was degraded in vitro and most of the membrane was degraded after 30 days	[154]
PEG	Hydrogels	PLGA–PEG–PLGA	Reduce inflammation, promote collagen deposition, and accelerate vascularization	-	[106]

## 4. Conclusions 

Various synthetic polymer-based wound dressings have been developed using a wide range of fabrication techniques such as hydrogel formation, electrospinning, solvent casting, freeze drying, 3D printing, etc. Due to the intricate dynamic condition of wound healing as well as the different types of wounds (sharp wounds, burns, and diabetic wounds), the selection of polymers and fabrication techniques should be depended on the specific requirements. Sometimes, the combination of two or more fabrication techniques can provide a multi-dimensional function to meet the complex needs of wound treatment. For example, wounds caused by burns are normally accompanied by exudation and severe pain. In such cases, hydrogel dressings can not only adsorb the exudate to keep the wound site dry but can also cool the wounds and relieve the pain of the patients [160]. In addition, anti-inflammatory substances or growth factors can be loaded in hydrogels to avoid infection and promote healing and are thus ideal candidates for burn dressings [30]. A diabetic wound is a chronic infectious wound with a strong inflammatory response associated [161]. Therefore, it is of great importance to endow the diabetic dressings with anti-inflammatory and antibacterial properties. In this regard, electrospun nanofibers and hydrogel materials can both protect the wounds from bacterial invasion and accelerate wound healing by loading anti-inflammatory and/or antibacterial drugs [162]. Especially due to the high glucose, acidic PH, and enhanced production of the reactive oxygen species (ROS) of the diabetic wounds, the development of wound dressing with glucose, PH, or ROS-responsive drug release would facilitate the outcomes of diabetic wound treatment [163].

Despite the many achievements with synthetic polymer-based dressing materials, most of them exhibit poor hydrophilicity and have fewer binding sites for cell adhesion and proliferation. In addition, they do not possess antibacterial or anti-inflammatory properties. Furthermore, some of the synthetic polymers such as PLGA and PU produced acidic degradation products which can cause adverse symptoms. Aiming at these problems, the combination of natural polymers with synthetic polymers and/or the delivery of drugs, growth factors, and anti-inflammatory or antibacterial substances would help to tailor these materials to acquire desirable features for skin tissue engineering applications.

Nowadays, wound dressings are moving towards intelligent, personalized, and high-end complex functions. For example, the use of nanodots with fluorescence and photothermal properties can enable accelerated wound healing and the real-time self-monitoring of dressing status [164]. The introduction of a high-precision personalized wound management model based on convolutional neural network (CNN) machine learning algorithm into wound dressings can ensure real-time monitoring, innovative analysis of the condition, and versatile management [165]. Furthermore, the wound dressing can be configured to match individual skin wounds by using intelligent algorithms [166]. Additionally, to mimic the complex structure of natural skin, wound dressing with bilayers to mimic the epidermis and dermis are recommended. Moreover, scarless healing treatments are also one of the important directions for the development of wound dressings in the future.

## Data Availability

Not applicable.
